# Generation of Amyloid-β Is Reduced by the Interaction of Calreticulin with Amyloid Precursor Protein, Presenilin and Nicastrin

**DOI:** 10.1371/journal.pone.0061299

**Published:** 2013-04-09

**Authors:** Nina Stemmer, Elena Strekalova, Nevena Djogo, Frank Plöger, Gabriele Loers, David Lutz, Friedrich Buck, Marek Michalak, Melitta Schachner, Ralf Kleene

**Affiliations:** 1 Zentrum für Molekulare Neurobiologie, Universitätsklinikum Hamburg-Eppendorf, Hamburg, Germany; 2 Institut für Klinische Chemie, Universitätsklinikum Hamburg-Eppendorf, Hamburg, Germany; 3 Department of Biochemistry, University of Alberta, Edmonton, Alberta, Canada; 4 Keck Center for Collaborative Neuroscience and Department of Cell Biology and Neuroscience, Rutgers University, Piscataway, New Jersey, United States of America; 5 Center for Neuroscience, Shantou University Medical College, Shantou, China; INSERM, UMR-S747, France

## Abstract

Dysregulation of the proteolytic processing of amyloid precursor protein by γ-secretase and the ensuing generation of amyloid-β is associated with the pathogenesis of Alzheimer's disease. Thus, the identification of amyloid precursor protein binding proteins involved in regulating processing of amyloid precursor protein by the γ-secretase complex is essential for understanding the mechanisms underlying the molecular pathology of the disease. We identified calreticulin as novel amyloid precursor protein interaction partner that binds to the γ-secretase cleavage site within amyloid precursor protein and showed that this Ca^2+^- and N-glycan-independent interaction is mediated by amino acids 330–344 in the C-terminal C-domain of calreticulin. Co-immunoprecipitation confirmed that calreticulin is not only associated with amyloid precursor protein but also with the γ-secretase complex members presenilin and nicastrin. Calreticulin was detected at the cell surface by surface biotinylation of cells overexpressing amyloid precursor protein and was co-localized by immunostaining with amyloid precursor protein and presenilin at the cell surface of hippocampal neurons. The P-domain of calreticulin located between the N-terminal N-domain and the C-domain interacts with presenilin, the catalytic subunit of the γ-secretase complex. The P- and C-domains also interact with nicastrin, another functionally important subunit of this complex. Transfection of amyloid precursor protein overexpressing cells with full-length calreticulin leads to a decrease in amyloid-β_42_ levels in culture supernatants, while transfection with the P-domain increases amyloid-β_40_ levels. Similarly, application of the recombinant P- or C-domains and of a synthetic calreticulin peptide comprising amino acid 330–344 to amyloid precursor protein overexpressing cells result in elevated amyloid-β_40_ and amyloid-β_42_ levels, respectively. These findings indicate that the interaction of calreticulin with amyloid precursor protein and the γ-secretase complex regulates the proteolytic processing of amyloid precursor protein by the γ-secretase complex, pointing to calreticulin as a potential target for therapy in Alzheimer's disease.

## Introduction

Alzheimer's disease (AD) is the most frequent form of dementia and its incidence rises with increasing life expectancy. Since the causes of AD are not fully understood, the elucidation of the molecular and cellular mechanisms underlying AD is of great importance. One of several hallmarks of AD pathology is the formation of amyloid plaques deriving from the amyloidogenic proteolysis of amyloid precursor protein (APP) [1]–[3], which is a transmembrane adhesion molecule of 695–770 amino acids [4]–[6]. In the amyloidogenic pathway, APP is cleaved by the β-secretase BACE [7], resulting in the generation of a soluble β-sAPP and the membrane bound C99 APP stump which is further cleaved by γ-secretase to generate the APP intracellular domain and amyloid-β (Aβ) peptides of different length ranging from 37 to 43 amino acids (Aβ_37_ to Aβ_43_) with Aβ_40_ as a major form. Alteration of the relative amounts of the individual Aβ peptides in the cerebrospinal fluid and blood correlates with the severity of AD pathology [2], [3], [8]–[10].

The γ-secretase is a transmembrane complex of at least four molecules: presenilin, nicastrin, presenilin enhancer 2 (PEN-2) und anterior pharynx defective 1 (APH-1) [11], [12]. Presenilin is the catalytic subunit of the complex. It undergoes autoproteolytic maturation, after which the resulting N-terminal and C-terminal fragments form a heterodimer. Nicastrin is a transmembrane glycoprotein and functions as a substrate receptor for proteins of various functions [13]. PEN-2 is required to stabilize the γ-secretase complex, while the function of APH-1 remains to be determined. After assembly of the functional γ-secretase complex in early compartments of the secretory pathway, the complex is transported to the plasma membrane and/or to late compartments of the secretory pathway [14]. In addition to its proteolytic activity, presenilin also exhibits a low, but functionally significant conductance for Ca^2+^ in the endoplasmic reticulum (ER), and many familial AD-associated presenilin mutations impair this function [15], indicating that presenilin functions as passive low conductance Ca^2+^ channel.

In the immature γ-secretase complex, presenilin forms a hydrophilic catalytic pore with an open conformational structure, while it adopts a conformation in the mature functional γ-secretase complex that forms a water-filled pore which provides the microenvironment for intramembranous cleavage of proteins [15]–[20]. Of particular importance for formation of such catalytic pore are the transmembrane domains TMD1, -7 and -9 of presenilin.

Calreticulin is a ubiquitously expressed soluble protein that displays multiple functions not only in intracellular compartments, such as the ER, cytoplasm and nucleus, but also in the extracellular space [21]–[26]. Its biological importance is revealed by embryonic lethality in mice when the calreticulin gene is ablated [27]. In the lumen of the ER, calreticulin functions as chaperone that is involved in protein quality control by elimination of proteins with improper folding, thus ensuring trafficking of proteins with proper folding and preventing protein aggregation [24], [28]. Calreticulin controls also metabolic and homeostatic Ca^2+^ levels in the cytosol and ER. Extracellular calreticulin, also called ecto-calreticulin, regulates diverse cellular activities, such as antigen processing and presentation, phagocytosis of apoptotic and cancer cells as well as cell adhesion, migration and proliferation [21]–[26], [29]. These different findings indicate that extracellular calreticulin regulates a multitude of physiological functions and underscore its critical impact in pathology when it is prevented to function normally. It is noteworthy in this context that calreticulin is found in a complex with APP and Aβ [30], [31] and that levels of the calreticulin mRNA and protein are reduced in patients with AD, suggesting that calreticulin is implicated also in the proteolytic processing of APP and, thus, in AD pathogenesis [32]. Since calreticulin binds to APP and Aβ, it is conceivable that calreticulin not only interacts with APP in intracellular compartments, but also at the plasma membrane and/or in extracellular compartments.

Here, we provide evidence that calreticulin interacts with APP and presenilin at the cell surface and that this interaction of calreticulin reduces the generation of Aβ.

## Materials and Methods

### Reagents and antibodies

Mouse monoclonal APP antibodies 22C11 and WO2 were from Chemicon (Hampshire, UK) and The Genetics Company (Schlieren, Switzerland), respectively. Rabbit polyclonal antibody B63.4 against the intracellular domain of APP was a kind gift from Bart De Strooper (University of Leuven, Belgium). Rabbit polyclonal antibody APP-ED against the extracellular domain was from GenWay Biotech (San Diego, CA, USA) and rabbit polyclonal antibodies against the N-terminus (A8967) or C-terminus (A8717) of APP or against actin were from Sigma-Aldrich (Saint Louis, MO, USA). Rabbit polyclonal antibody against calmodulin and goat polyclonal antibodies against the N-terminus (T-19, sc-7431) or the C-terminus (C-17; sc-6467) of calreticulin were from Santa Cruz Biotechnology (Heidelberg, Germany). Rabbit calreticulin antibody CRT283 and rabbit L1 antibody were described [33], [34]. Rabbit monoclonal antibody `Nixoń (with the consent of Ralph Nixon, Nathan S. Kline Institute for Psychiatric Research, Orangeburg, NY, USA) and rabbit polyclonal antibody 2953 against presenilin-1 as well as the rabbit polyclonal antibody N1660 raised against the C-terminus of nicastrin and the rabbit polyclonal 2D8 antibody directed against Aβ were kindly provided by Harald Steiner (Ludwig-Maximilians-Universität München, Germany). Secondary antibodies and control antibodies (non-immune serum or IgG) were from Dianova (Hamburg, Germany). Synthetic biotinylated APP-γ (biotin-SNKGAIIGLMVGGVVIATVIVITLVMLKKKC-OH) and APP-β (biotin-TNIKTEEISEVKMDAEFGHDSGFEVRHQKC-OH) peptides as well as the calreticulin peptide (FLITNDEAYAEEFGN) were from Schafer-N (Copenhagen, Denmark). Antisense peptide NH_2_-QGDDDHCRYDNTAHH-OH was synthesized by Chirion Technologies (Clayton, Australia) or Schafer-N.

### Recombinant calreticulin proteins and calreticulin cDNA constructs

Recombinant rabbit calreticulin as well as glutathione S-transferase (GST) fusion proteins comprising full-length calreticulin or calreticulin domains N and P (amino acid 1–290), P (amino acid 182–290) or C (amino acid 330–401) were prepared as previously described [35]. The cDNA encoding full-length rabbit calreticulin, the N-domain (amino acid 1–182), the P-domain (amino acid 182–290), or the N- and P-domains (amino acid 1–290) of calreticulin were generated as previously described [36]. The cDNA encoding N- and P-domains of calreticulin were subcloned into the pcDNA3/Myc-His plasmid via EcoRI/XhoI. The SPA4CT plasmid (APP-C99, myc-tag) was a kind gift from Stefan Kins (ZMBH, Heidelberg, Germany).

### Generation of antisense antibody

Two mg antisense peptide and 2 mg keyhole limpet hemocyanin (Merck, Darmstadt, Germany) were incubated in 500 µl 0.1 M phosphate buffer, pH 7.3, with 0.5% glutaraldehyde for 30 min at room temperature. After addition of 100 µl 1 M glycine, pH 6.0, and 1.5 ml phosphate-buffered saline, pH 7.3 (PBS), the solution was used to immunize rabbits (2 boosts, 500 µl per boost). For isolation of the IgG fractions the DEAE Affi-Gel Blue Gel kit (Bio-Rad, München, Germany) was used according to the manufacturer's instructions. Briefly, DEAE Affi-Gel Blue gel (7 ml gel per ml serum) was washed with 0.1 M acetic acid, pH 3.0, containing 1.4 M NaCl and with 40% (v/v) isopropanol and equilibrated with sample buffer (20 mM Tris-HCl, pH 8.0, 28 mM NaCl, 0.02% NaN_3_). Serum dialyzed against sample buffer was applied and after washing with sample buffer, IgG proteins were eluted with 0.1 M citrate buffer, pH 3.0 and immediately adjusted to pH 7.3 using NaOH.

### Synaptosomal membrane preparation

Brains were prepared from adult C57BL/6J mice and transferred to a Potter homogenizer (Teflon pestle, 0.1 µm from Novodirect, Kehl, Germany). All following steps were carried out at 4°C. Brains were homogenized in 3 ml of Tris-plus buffer (5 mM Tris-HCl, pH 7.4, 1 mM CaCl_2_ and 1 mM MgCl_2_) containing 0.32 M sucrose. The homogenate was centrifuged at 1,400 g for 10 min and the resulting supernatant was centrifuged at 17,500 g for 15 min. The 1,400 g and 17,500 g pellet were resuspended in Tris-plus buffer containing 0.32 M sucrose and applied to a sucrose step gradient (1.2 M, 1.0 M, 0.85 M and 0.65 M sucrose in Tris-plus buffer). All following centrifugations were carried out at 100,000 g. The sucrose gradients were centrifuged for 1 h and the material at the 1.0/1.2 M interfaces was diluted with Tris-plus buffer and collected by centrifugation for 30 min. The pelleted material from the 1.0/1.2 M interfaces contained synaptosomes. The combined subfractions were resuspended in Tris-plus buffer, incubated for 30 min and centrifuged for 20 min. The pellet was then resuspended in Tris buffer (5 mM Tris-HCl, pH 7.4) and applied to a sucrose step gradient (1.2 M, 1.0 M, 0.85 M and 0.65 M sucrose in Tris buffer). The gradient was centrifuged for 1 h and the material from the 1.0/1.2 M interface containing synaptosomal membranes was collected by centrifugation for 30 min. The pellets were incubated in alkaline buffer (0.15 M NaHCO_3_, pH 10.0, 5 mM EDTA) for 30 min and applied to a sucrose step gradient (1.2 M, 1.0 M, 0.85 M and 0.65 M in alkaline buffer). After centrifugation for 1 h, the material at the 1.0/1.2 M interface containing synaptosomal membranes depleted of membrane-associated peripheral proteins were collected by centrifugation for 30 min. The pellet was subjected to consecutive solubilization by 1% Triton-X100 and 1%, 2% and 5% N-octyl β-D-glucopyranoside. In each solubilization step, pellets were resuspended in Tris-buffer, incubated for 1 h in the presence of detergent and centrifuged at 15,000 g for 30 min. All supernatants containing detergent-soluble proteins were collected and used for immunoaffinity chromatography.

### SDS-polyacrylamide gel electrophoresis and Western blot analysis

Samples were boiled in SDS sample buffer (80 mM Tris/HCl, pH 6.8, 10% glycerol, 1% SDS, and 5% mercaptoethanol or 0.5% dithiothreitol) and subjected to SDS-PAGE. Proteins were transferred onto nitrocellulose membranes (Schleicher and Schüll, Dassel, Germany), which were then incubated for 1 h at room temperature in blocking buffer consisting of PBS, pH 7.4, and 5% skim milk powder, washed three times with PBS, and incubated overnight at 4°C with primary antibodies. After washing three times with PBS containing 0.05% Tween-20 (PBST), membranes were incubated for 1 h with horseradish peroxidase (HRP) conjugated secondary antibodies in blocking buffer. After five washes with PBST, enhanced chemiluminescence detection was carried out using SuperSignal (Pierce, Rockford, IL, USA).

### Immunoaffinity chromatography

Coupling of purified IgG fractions to CarboLink columns (Pierce) and subsequent affinity purification steps were performed according to the manufacturer's instructions. One ml of each sample was loaded onto CarboLink columns with immobilized purified IgG (4–8 mg) and incubated for 60 min at room temperature. After several washing steps bound proteins were eluted with 0.1 M glycine, pH 2.5, and immediately neutralized by 1 M Tris-HCl, pH 9.5. The eluate was dialyzed against PBS containing 0.1% of N-octyl β-D-glucopyranoside.

### Mass spectrometry

Protein samples were subjected to SDS-PAGE followed by staining with the colloidal Coomassie blue staining Roti-Blue kit (Carl Roth, Karlsruhe, Germany) and stained protein bands were cut out of the gel. After successive treatment with dithiothreitol and iodoacetamide, in-gel digestion of proteins by 5 ng trypsin/ µl (Promega, Mannheim, Germany) in 50 mM NH_4_HCO_3_ was carried out overnight at 37°C. Gel pieces were then repeatedly extracted with 50% acetonitrile/5% formic acid, and the combined extracts were dried down in a vacuum concentrator, re-dissolved in 5% methanol/5% formic acid, desalted on a C18 µZipTip (Millipore, Schwalbach, Germany), eluted with 1 µl 60% methanol/5% formic acid and analyzed by nano-electrospray mass spectrometry in a QTOF II instrument (Micromass, Manchester, UK). MS/MS spectra obtained by collision induced fragmentation after manual precursor selection were evaluated by the Mascot MS/MS search algorithm version 2.2 (Matrix Sciences, London, UK) using the following parameters: precursor mass tolerance: 1.4 Da, fragment mass tolerance: 0.6 Da, one missed tryptic cleavage allowed, fixed modification: carbamidomethyl cysteine, variable modification: monooxidized methionine, databases searched: NCBInr 20090508 and SwissProt 57.2. Searches were limited to the species *Mus musculus* (Mascot probability based MOWSE score significance threshold for p<0.05: 20).

### Cell culture and transfection

CHO cells (CHO-K1; ATCC #CCL-61) stably transfected with APP were grown in F-12 medium containing 10% fetal calf serum (FCS), 1% sodium pyruvate, 2% glutamine and hygromycine (0.8 mg/ml). HEK cells stably transfected with the Swedish mutant of APP and either with wild type presenilin-1 (WT-23) or presenilin-1 mutated in the catalytically active site (D385N-10), kindly provided by Harald Steiner, were grown in DMEM medium supplemented with 10% FCS, 1% sodium pyruvate, 2% glutamine, G418 (200 µl/ml) and zeocin (200 µl/ml; Invitrogen, Karlsruhe, Germany).

For transfection of confluent cells, the Lipofectamine Plus kit (Invitrogen) was used. One day before transfection, the cells were seeded in 6-well-plates. When the cell density had reached 70–80% confluence, cells were washed with PBS and transfected with 1 µg DNA. The transfection was performed as described in the manufacturer's protocol and was terminated after 3 h by addition of medium. Recombinant calreticulin proteins were added to cultured neurons at a concentration of 1.5 µM. Cell surface biotinylation was carried out as described [34]. Briefly, cells were washed twice with ice-cold PBS-2+ (PBS, 0.5 mM CaCl_2_, 2 mM MgCl_2_) and incubated for 10 min on ice with 0.5 mg of sulfo-NHS-LS-biotin (Pierce) freshly dissolved in PBS-2+. After treating the cells with 20 mM glycine in PBS-2+ for 5 min on ice, cells were washed twice with ice-cold PBS-2+ and lysed with RIPA buffer (50 mM Tris, 150 mM NaCl, pH 7.4, 1% NP40) for 30 min at 4°C with mild shaking. Lysed cells were collected using a rubber scraper and centrifuged for 10 min at 1,000 g and 4°C. Streptavidin-coupled magnetic beads (Invitrogen) were added to the lysate and incubated overnight at 4°C. After washing of magnetic beads, proteins were eluted by boiling in SDS sample buffer and subjected to Western blot analysis.

### Immunoprecipitations and pull-down assay

Cultured cells were washed three times with cold PBS, and then solubilized at 4°C for 30 min using RIPA buffer supplemented with complete protease inhibitor cocktail (Roche, Mannheim, Germany). Synaptosomes were incubated in the presence of 1% N-octyl β-D-glucopyranoside for 40 min at 4°C. For immunoprecipitation, the samples were centrifuged at 1,000 g and the resulting supernatants were incubated with antibodies for 3 h at 4°C. Fifty µl Protein A/G-suspension (Santa Cruz Biotechnology) were added to the mixture and incubated overnight at 4°C. Beads were pelleted at 1,000 g and 4°C, washed three times with ice-cold PBS containing 1% N-octyl β-D-glucopyranoside, Triton-X100 or CHAPS for 10 min, and once with PBS. SDS sample buffer was added to the beads and the samples were boiled at 95°C for 5 min. The beads were pelleted by centrifugation and the supernatants were subjected to SDS-PAGE.

Purified GST or the GST fusion proteins (100 µg) were bound to glutathione-agarose beads (Santa Cruz Biotechnology) and incubated with cell lysates or the respective membrane fractions overnight at 4°C on a rocking platform. Beads were pelleted at 1,000 g, washed three times with lysis buffer and once with PBS at 4°C. Bound proteins were eluted by incubation with 40 mM glutathione.

### Binding assays

For ELISA, proteins or peptides were immobilized on a polyvinylchloride surface in 96-well-plates (Nunc, Roskilde, Denmark) at concentrations of 5–10 µg/ml overnight at 4°C. The following steps were carried out at room temperature. After washing five times with PBS, wells were blocked by adding 2% BSA in PBS (PBS/BSA) for 1 h. After washing three times with PBS containing 0.05% Tween-20 (PBS-T), proteins or peptides in PBS-T containing 1% BSA, 1 mM CaCl_2_, 1 mM MgCl_2_ and 1 mM MnCl_2_ were added at different concentrations and incubated for 1 h. The plates were washed five times and bound proteins were detected with HRP coupled streptavidin or primary antibodies and HRP-coupled secondary antibodies. Freshly prepared staining solution of 2% 2,2′-azino-bis[3-ethylbenzthiazoline-6-sulphonic acid] in 100 mM sodium acetate buffer, pH 4.2, and 0.001% H_2_O_2_ was added and the reaction was stopped by the addition of 0.6% SDS solution in water. Bound conjugates were quantified by measuring the absorbance at 405 nm using an ELISA reader.

Label-free binding assay using BIND Technology (SRU Biosystems) was performed as described [34]. A 384-well plate with a TiO_2_ biosensor surface (SRU Biosystems) was used for substrate coating. Different concentrations of soluble binding partners were applied and the peak wavelength shift of reflected light was measured. The change in peak wavelength is proportional to the binding of proteins to the plate surface or to the immobilized target molecules. If not stated otherwise, washing and blocking conditions were the same as for the ELISA experiments.

### Immunocytochemistry of hippocampal neurons

Hippocampal cultures were prepared from 2-day-old C57BL/6J mice as described [34], [37] and maintained on poly-L-lysine-coated coverslips for one day or one week. For live cell staining, primary antibodies were incubated with live cells for 15 min on ice. After washing three times with PBS and blocking with 2% BSA in PBS for 1 h at room temperature, cells were washed three times with PBS and either incubated with Cy2-labeled secondary rabbit antibody for 1 h at room temperature in the dark or subjected to fixation with 4% paraformaldehyde in PBS for 10 min at room temperature. Fixed cells were washed twice with PBS and blocked with 1% BSA in PBS for 30 min at 4°C. After blocking, cells were washed three times with PBS and incubated overnight without or with primary antibodies at 4°C followed by three washes with PBS and incubation with Cy2- and/or Cy3-labeled secondary antibodies for 1 h at room temperature in the dark. Finally, cells were washed three times with PBS, mounted with Aqua Poly-Mount medium (Polysciences, Eppenheim, Germany) and analyzed using a Zeiss LSM510 confocal laser-scanning microscope (60× oil-immersion objective lens). Images were scanned with a resolution of 512×512 pixels. Detector gain and pinhole were adjusted to give an optimal signal-to-noise ratio.

### Quantification of the Aβ_40_ and Aβ_42_ peptides by ELISA

Levels of Aβ_40_ and Aβ_42_ in conditioned medium were quantified using Amyloid β40 and Amyloid β42 ELISA kits (The Genetics Company, Schlieren, Switzerland) or Human/Rat β Amyloid (40) ELISA kit and Human/Rat β Amyloid (42) ELISA kit (Wako Chemicals, Richmond, VA, USA) as described in the manufacturer's protocol.

### Immuno-isolation of the γ-secretase complex

For isolation of intact γ-secretase complexes, HEK cells stably transfected with mutated APP and wild type or mutated presenilin-1 (D385N) were used. Cells were homogenized at 4°C in MOPS buffer (10 mM 3-(N-morpholino)propanesulfonic acid, pH 7.0, 10 mM KCl) containing protease inhibitor cocktail (Roche). After centrifugation for 10 min at 1,000 g and 4°C, the supernatant was further centrifuged for 45 min at 100,000 g. The pelleted membranes were incubated with CHAPSO lysis buffer (1% 3-[(3-cholamidopropyl)dimethylammonio]-2-hydroxy-1-propanesulfonate, pH 6.4, 150 mM sodium citrate) containing protease inhibitor cocktail for 30 min on ice and centrifuged for 30 min at 100,000 g and 4°C. After preclearing, the supernatant was incubated with protein A/G agarose and the monoclonal presenilin-1 antibody `Nixoń for 2 h at 4°C. Immunoprecipitates were obtained by centrifugation at 2,000 g for 5 min and 4°C, washed 4 times with CHAPSO-wash buffer (0.5% CHAPSO, pH 6.4, 150 mM sodium citrate) containing protease inhibitor cocktail, and resuspended in 0.2% CHAPSO, 35 mM sodium citrate, pH 6.4, 3.5% glycerol, 30 mM DTT, 0.5 mg/ml L-α-phosphatidylcholine (Sigma, Taufkirchen, Germany), protease inhibitor cocktail (Roche).

### *In vitro* γ-secretase activity assay

The assay was carried out according to a protocol recently described [38]. Briefly, after immunoprecipitation, the immuno-isolated γ-secretase was incubated with 0.25 µM purified recombinant C100-His6 substrate, kindly provided by Harald Steiner, and 0.25 µM GST fusion proteins with full-length calreticulin or the P-domain of calreticulin or GST overnight at 37°C. The reaction was stopped by adding sample buffer and the amount of produced Aβ was analyzed by Western blot analysis.

## Results

### Calreticulin interacts with APP

**Figure 1:**
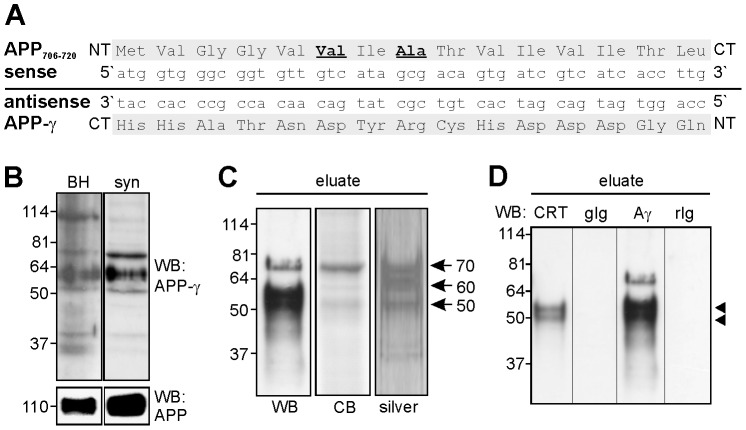
Identification of APP-interacting proteins using an antibody generated against a peptide complementary to the γ-cleavage site of APP. (A) The DNA sequence of the sense strand coding for the amino acids 706–720 of APP comprising the γ-cleavage site for generation of Aβ_40_ (bolded Val) and Aβ_42_ (bolded Ala) and the corresponding antisense DNA strand and the corresponding antisense peptide (APP-γ) are shown (N-terminus, NT; C-terminus, CT). (B) Western blot analysis of homogenate (BH) and synaptosomal fraction (syn) from mouse brain using the antibody directed to the antisense peptide (APP-γ; upper panel) or the APP antibody 22C11 (lower panel). (C) Proteins isolated from synaptosomal membranes by immunoaffinity chromatography using APP-γ antibody (eluate) were subjected to Western blot analysis using APP-γ antibody (WB) and to silver or Coomassie blue (CB) staining. Arrows indicate the position of the immuno-purified protein bands with apparent molecular weights of approximately 50, 60, 70 kDa. (D) The eluate from the APP-γ column (eluate) was subjected to Western blot analysis using the goat calreticulin antibody C-17 (CRT), the rabbit antibody APP-γ (A γ) directed to the antisense peptide and goat (gIg) or rabbit (rIg) non-immune control antibodies. Arrowheads indicate a double band of calreticulin with an apparent molecular weight of 50–55 kDa.

To identify proteins involved in the cleavage of APP we used the concept of complementary hydropathy [39]–[45]. According to the theory of complementary hydropathy, an antisense peptide should exhibit sequence similarity and/or structural features of proteins interacting with functionally important sense peptide stretches. We thus generated an antibody against an antisense peptide which is complementary to the amino acid sequence of APP at the γ-cleavage site (Fig. 1A) for isolation and identification of proteins that interact with the γ-cleavage site within APP. By Western blot analysis, this antibody recognized several bands with different apparent molecular weights in detergent lysates of adult mouse brain tissue (Fig. 1B). In a synaptosomal fraction, the antibody recognized three major bands of approximately 50, 60 and 70 kDa (Fig. 1B). Since the synaptosomal fraction was enriched in APP (Fig. 1B) and in three proteins which reacted with the antisense peptide antibody, this fraction was used for isolation of potential APP binding proteins. To obtain synaptosomal membranes enriched in proteins that were tightly associated with or embedded in the membranes, synaptosomal membranes were treated at alkaline pH in the presence of EDTA to remove membrane-associated peripheral proteins. After treating these membranes successively with Triton-X100 and increasing concentrations of N-octyl β-D-glucopyranoside, detergent-solubilized proteins were pooled and subjected to immunoaffinity chromatography using immobilized antisense peptide antibody. Immunoaffinity purified proteins were immunostained with the antibody against the antisense peptide as well as visualized by silver or Coomassie blue staining (Fig. 1C). The three major bands with apparent molecular weights of approximately 50, 60 and 70 kDa seen by immunostaining as well as by silver and Coomassie staining were analyzed by mass spectrometry. After tryptic digestion of the 50 kDa band, one of the detected peptides could be assigned to calreticulin by nano-electrospray mass spectrometry. The MS/MS spectrum of a 1451.35 Da mass (detected as double charged ion at m/z = 726.68) matched the tryptic peptide EQFLDGDAWTNR of calreticulin (MOWSE score: 30). All other assigned peptides in the tryptic digest of the 50 kDa band could be assigned either to the antibody used or to contaminating keratins.

Western blot analysis of the eluate revealed that a double band of 50–55 kDa was recognized by a polyclonal antibody against calreticulin and the antibody against the antisense peptide which recognized an additional band of approximately 70 kDa, while non-immune control antibodies did not detect either band (Fig. 1D). This result indicated that the immunopurified 50 kDa protein is calreticulin.

### Calreticulin binds to the γ-cleavage site of APP and not to N-glycans on APP

**Figure 2:**
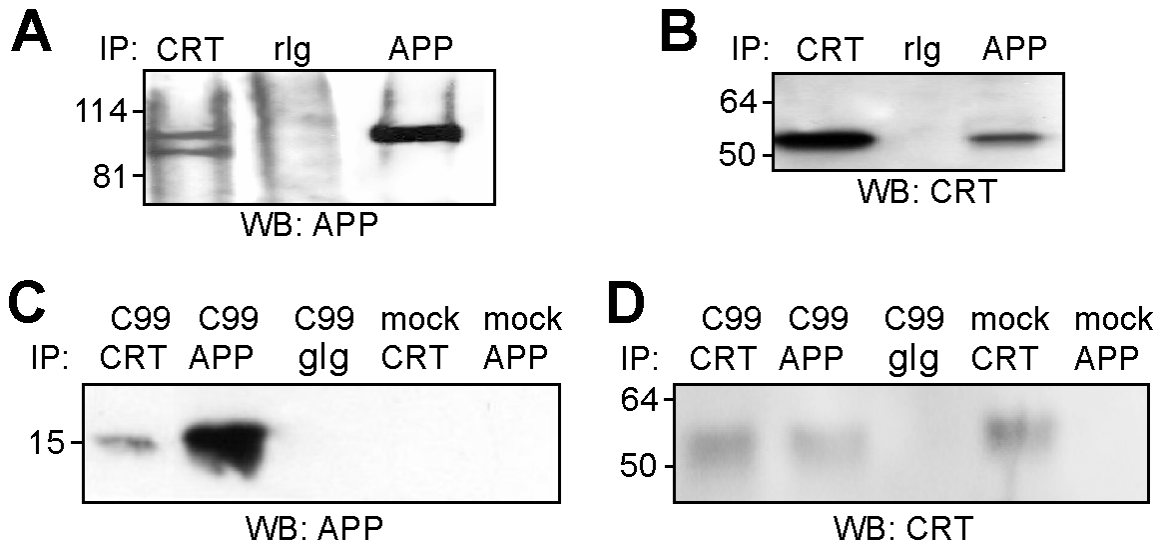
Co-immunoprecipitation of calreticulin and APP. (**A, B**) A synaptosomal fraction was subjected to immunoprecipitation using the rabbit antibody CRT283 against calreticulin (CRT) (**A, B**), the rabbit APP antibody WO2 (APP) (**A**) or B63-4 (**B**) or non-immune control rabbit antibody (rIg) (**A, B**). (**C**) CHO cells were mock-transfected or transfected with a construct encoding the C99 APP stump and used for immunoprecipitation with the goat calreticulin antibody C-17 (CRT), the rabbit APP antibody ED-APP (APP) or non-immune control goat antibody (gIg). (**A, B, C**) Immunoprecipitates were subjected to Western blot analysis using the APP antibody 22C11 (**A**), the goat calreticulin antibody C-17 (**B**) or the rabbit polyclonal APP antibody B63–4 recognizing the C99 APP stump (**C**).

The interaction between calreticulin and APP was further investigated by immunoprecipitation using a detergent extract of synaptosomes isolated from mouse brain. Western blot analysis using a mouse monoclonal APP antibody showed that two APP forms with apparent molecular weights of approximately 100 and 110 kDa were present in the immunoprecipitate when a rabbit polyclonal calreticulin antibody was used for immunoprecipitation (Fig. 2A). These bands were not detected when a non-immune rabbit control antibody was used for immunoprecipitation (Fig. 2A). Only the 110 kDa form of APP which represents the mature and fully glycosylated APP [46] was detectable in the immunoprecipitate when a rabbit polyclonal APP antibody was used for immunoprecipitation as positive control, while the 100 kDa form representing immature APP [46] was not detectable (Fig. 2A). Western blot analysis with a goat calreticulin antibody showed one protein band with an apparent molecular weight of approximately 50 kDa when a rabbit APP antibody was used for immunoprecipitation (Fig. 2B). A similar band was observed in the immunoprecipitate with a rabbit calreticulin antibody, while no such band was detectable when non-immune rabbit control antibody was used for immunoprecipitation (Fig. 2B). In summary, co-immunoprecipitations indicate that mature APP and calreticulin are associated.

Since calreticulin is a lectin recognizing core-glycosylated N-glycans [47], [48], it was conceivable that the interaction between calreticulin and APP is due to the binding of calreticulin to N-glycans carried by APP. To exclude this possibility, CHO cells transfected with a construct encoding the C99 APP stump, which does not carry N-glycans, were used for immunoprecipitations. C99 was detectable as a protein with an apparent molecular weight of approximately 15 kDa by Western blot analysis with a C99-recognizing APP antibody when a calreticulin or APP antibody was used for immunoprecipitation from cells expressing C99, while it was not detectable in APP or calreticulin immunoprecipitate from mock-transfected cells (Fig. 2C). No immunoreactive protein band was detectable when a non-immune control antibody was used for immunoprecipitation from lysates of cells expressing C99 (Fig. 2C). Western blot analysis with a calreticulin antibody showed calreticulin in the calreticulin immunoprecipitates from mock-transfected cells and cells expressing C99 as well as in APP immunoprecipitates from C99-expressing cells (Fig. 2D). Calreticulin was not detectable in APP immunoprecipitates from mock-transfected cells or in immunoprecipitates from cells expressing C99 when a non-immune control antibody was used for immunoprecipitation (Fig. 2D). These results indicate that the interaction of calreticulin and APP does not depend on APP-associated N-glycans.

**Figure 3:**
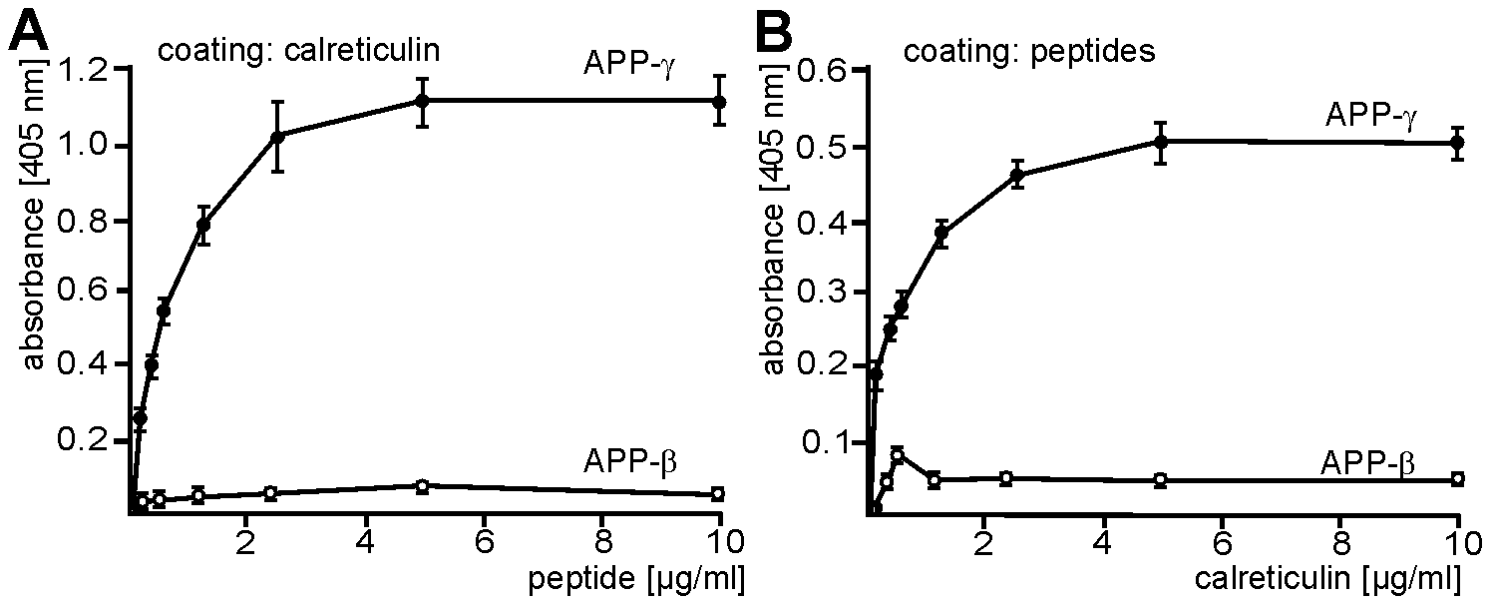
Binding of calreticulin to the synthetic peptide containing the γ-cleavage site of APP. Recombinant calreticulin (**A**) or synthetic biotinylated peptides comprising the γ-secretase cleavage site (APP- γ) or the β-secretase cleavage site (APP-β) (**B**) were coated as substrates and incubated with different concentrations of biotinylated APP-γ or APP-β peptides (**A**) or recombinant calreticulin (**B**). Detection of bound proteins was carried out using HRP-coupled streptavidin (**A**) or the rabbit calreticulin antibody CRT283 and HRP-coupled secondary antibodies (**B**). Mean values ± SD from three independent experiments are shown.

To test for the direct binding of calreticulin to the γ-cleavage site of APP, we performed an ELISA-based binding assay with recombinant calreticulin and synthetic biotinylated peptides comprising either the γ-cleavage site of APP or the β-cleavage site of APP as control. The peptide containing the γ-cleavage site showed concentration-dependent and saturable binding to substrate-coated calreticulin, while the control peptide did not bind to calreticulin (Fig. 3A). When using these peptides as substrate coats, concentration-dependent and saturable binding of soluble calreticulin to the peptide comprising the γ-cleavage site, but not to the control peptide, was observed (Fig. 3B). These experiments show that calreticulin and APP directly interact and that calreticulin binds to a sequence stretch within the transmembrane domain of APP that carries the γ-secretase cleavage site.

### APP interacts with the C-domain of calreticulin in a Ca^2+^-independent manner

**Figure 4:**
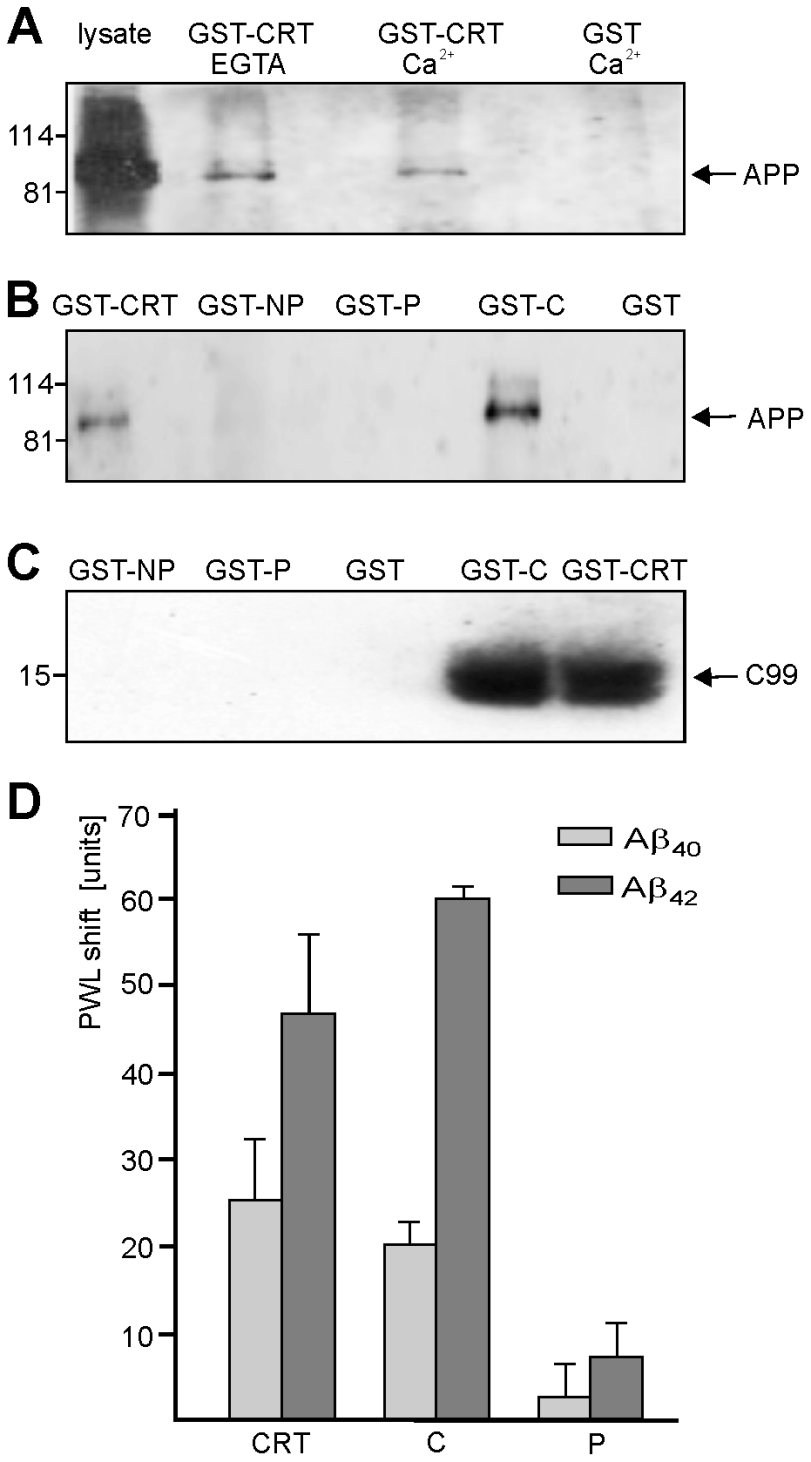
Pull-down of APP with GST-calreticulin fusion proteins and binding of calreticulin to Aβ_40_ and Aβ_42_. GST or GST-fusion proteins comprising full length calreticulin (GST-CRT) (**A-C**), N- and P-domains (GST-NP), P-domain (GST-P) or C-domain (GST-C) (**B, C**) were bound to glutathione-agarose beads and incubated with lysates of APP overexpressing CHO cells in the presence of Ca^2+^ or EGTA (**A**) or the absence of Ca^2+^ or EGTA (**B**) or were incubated with lysates of CHO cell expressing the C99 APP stump (**C**). Precipitated proteins were subjected to Western blot analysis using the APP antibody 22C11 (**A, B**) or the rabbit APP antibody B63-4 recognizing the C99 APP stump (**C**). (**D**) Label-free binding assay using substrate-coated GST-CRT (CRT), GST/C-domain (C) or GST/P-domain (P) and soluble Aβ_40_ or Aβ_42_ peptides. Mean values ± SD from triplicates of a representative experiment are shown. The experiment was repeated once with identical results.

The interaction between APP and calreticulin was further characterized in a pull-down approach. Since calreticulin is a Ca^2+^-binding chaperone we tested whether binding of calreticulin to APP was Ca^2+^-dependent. A GST-fusion protein containing full-length calreticulin (GST/calreticulin) was incubated with a lysate of APP overexpressing CHO cells in the presence of Ca^2+^ or the Ca^2+^ chelator EGTA to deplete Ca^2+^. APP was pulled down equally well under both conditions by GST/calreticulin, but not by GST even in the presence of Ca^2+^ (Fig. 4A), indicating that the interaction between APP and calreticulin is Ca^2+^-independent.

Since calreticulin consists of three well-characterized domains: the N-terminal N-domain, the central P-domain, and the C-terminal C-domain [49], [50], we addressed the question which domain of calreticulin mediates the binding to APP. GST-fusion proteins comprising all three domains (GST/calreticulin), the C-domain (GST/C-domain), the P-domain (GST/P-domain), or the N- and P-domain (GST/NP-domain) were used for pull-down assays with lysates of CHO cells overexpressing full-length APP or the C99 APP stump. GST/calreticulin and GST/C-domain pulled down full-length APP and C99 (Fig. 4B, C). No APP or C99 was detectable when the GST/P-domain, GST/NP-domain or GST was applied (Fig. 4B, C). The results indicate that the interaction of calreticulin and APP is mediated by the C-domain of calreticulin.

To test whether calreticulin also binds via its C-domain to Aβ_40_ and/or to Aβ_42_, we determined the binding of GST/calreticulin and GST/C-domain to substrate-coated Aβ_40_ or Aβ_42_ in a label-free binding assay. The GST/P-domain, which did not bind to APP, was used as negative control. The GST/calreticulin and GST/C-domain bound to substrate-coated Aβ_40_ and Aβ_42_, while GST/P-domain neither bound to Aβ_40_ nor to Aβ_42_ (Fig. 4D). This result indicates that calreticulin also interacts with Aβ_40_ and Aβ_42_ via its C-domain.

### APP and calreticulin interact at the cell surface

**Figure 5:**
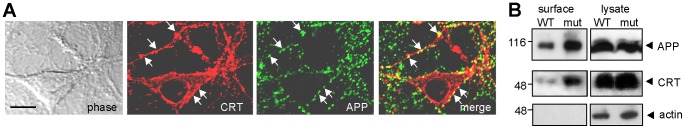
Co-localization of APP and calreticulin in primary cultures of live hippocampal neurons. (**A**) Hippocampal neurons maintained for one week in culture were incubated with the rabbit APP antibody WO2 (green). After fixation, the neurons were immunostained with the goat calreticulin antibody C-17 (red). Superimposition of immunostainings (merge) shows co-localization of calreticulin and APP (yellow). Phase contrast shows the cellular structures. Scale bar, 10 µm. (**B**) Cell surface biotinylation of HEK cells overexpressing wild-type (WT) or mutated (mut) presenilin. Biotinylated surface proteins were isolated and subjected to Western blot analysis with the rabbit APP antibody B63-4, the goat calreticulin antibody C-17 (CRT) or an actin antibody. Cell lysates were used as input control.

Next, we asked in which compartment APP and calreticulin interact and analyzed the localization of both proteins in primary cultures of hippocampal neurons. Live neurons were incubated first with a rabbit polyclonal APP antibody followed by green-fluorescent labeled secondary rabbit antibodies before fixation. Fixed neurons were then incubated with a goat calreticulin antibody and red-fluorescent labeled secondary goat antibody. Co-immunostaining was seen at the cell surface of cell bodies and neurites (Fig. 5A), indicating that calreticulin and APP co-localize at the plasma membrane.

To support the notion that calreticulin is present in the extracellular compartment, cell surface biotinylation of live cells overexpressing APP was performed, since APP and calreticulin are not abundant enough at the cell surface of cultured hippocampal neurons to perform cell surface biotinylation. When using HEK cells overexpressing wild-type or mutated presenilin, the amounts of both biotinylated APP and calreticulin at the cell surface relative to total amounts, being similar in all cell lysates (Fig. 5B), were approximately 2- to 3-fold higher in cells overexpressing the mutated presenilin when compared to cell overexpressing wild-type presenilin (Fig. 5B). This result indicates that APP and calreticulin accumulate at the cell surface of cells overexpressing mutated presenilin, and the concomitant accumulation implies that APP and calreticulin either form a complex at the cell surface or reach the cell surface as a complex. Importantly, cytoplasmic actin was not detectable as biotinylated protein, showing that cells remained intact during the biotinylation reaction (Fig. 5B).

**Figure 6:**
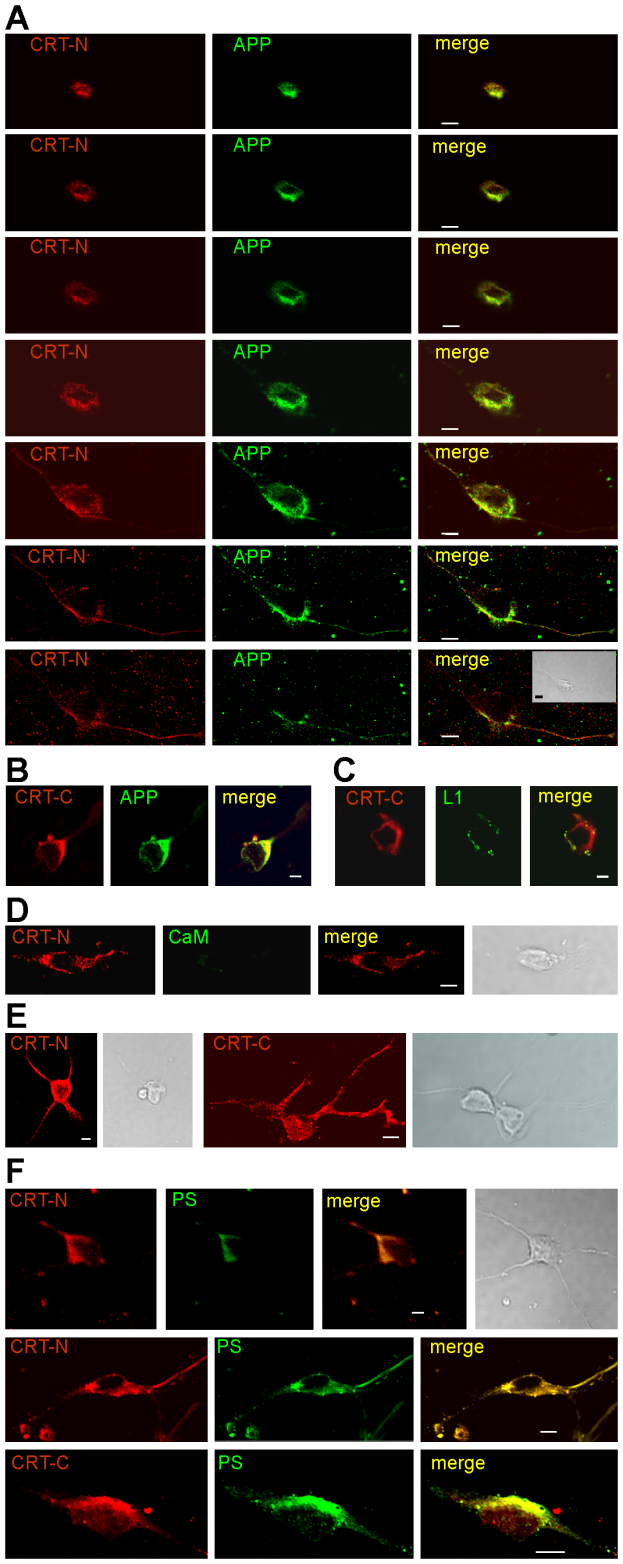
Co-staining of calreticulin with APP and presenilin at the cell surface of live hippocampal neurons. Live hippocampal neurons were incubated with goat calreticulin antibody T-19 recognizing the N-terminus (CRT-N) (**A, D, F**) or C-17 recognizing the C-terminus (CRT-C) (**B, C, F**) and with rabbit APP antibody A8967 directed against the N-terminus (**A, B**), rabbit L1 (**C**) or calmodulin (CaM) (**D**) antibody. After fixation, cells were incubated with the calreticulin antibodies (**E**) or the rabbit presenilin antibody 2953 and fluorescent-labeled secondary antibodies. Superimposition of immunostainings (merge) shows co-localization of calreticulin and APP (yellow). Phase contrast shows the cellular structures. Scale bar, 5 µm.

To investigate whether calreticulin and APP interact at the cell surface of hippocampal neurons and whether calreticulin is located in the extracellular compartment, live cultured hippocampal neurons were incubated with rabbit antibody against APP and goat antibody against the N-terminus of calreticulin, fixed and stained with fluorescent labeled rabbit and goat secondary antibodies. Confocal scanning showed immunostaining of calreticulin and APP at the top of a cell body, followed by a ring-like staining in the consecutive sectional layers of the cell body and staining of neurites in the lowest layer (Fig. 6A). A pronounced overlap of APP and calreticulin immunostaining was observed in all layers (Fig. 6A). Similarly, overlapping cell surface immunostaining was also seen with the APP antibody and an antibody directed against the C-terminus of calreticulin (Fig. 6B). Live staining using antibodies against the cell adhesion molecule L1 and calreticulin showed cell surface staining of calreticulin and L1, but no co-staining of L1 and calreticulin (Fig. 6C). When using a calmodulin antibody and the calreticulin antibodies for live staining, a pronounced cell surface immunostaining for calreticulin was found, but no immunostaining for calmodulin was detectable (Fig. 6D). A strong punctate intracellular staining was observed with both calreticulin antibodies when they were applied to fixed neurons (Fig. 6E). The findings that the cytoplasmic calmodulin and the intracellular pool of calreticulin were not detectable upon live staining confirmed the intactness of the neurons during live staining. The co-localization of calreticulin and APP, but not L1 indicates the specificity of the calreticulin and APP interaction at the plasma membrane. The immunostaining at the cell surface obtained with the antibodies recognizing either the N- or the C-terminus of calreticulin indicates that both the N- and C-terminus are accessible from or exposed to the extracellular side of the plasma membrane and/or that calreticulin is present in the extracellular space.

### Calreticulin reduces the generation of Aβ

**Figure 7:**
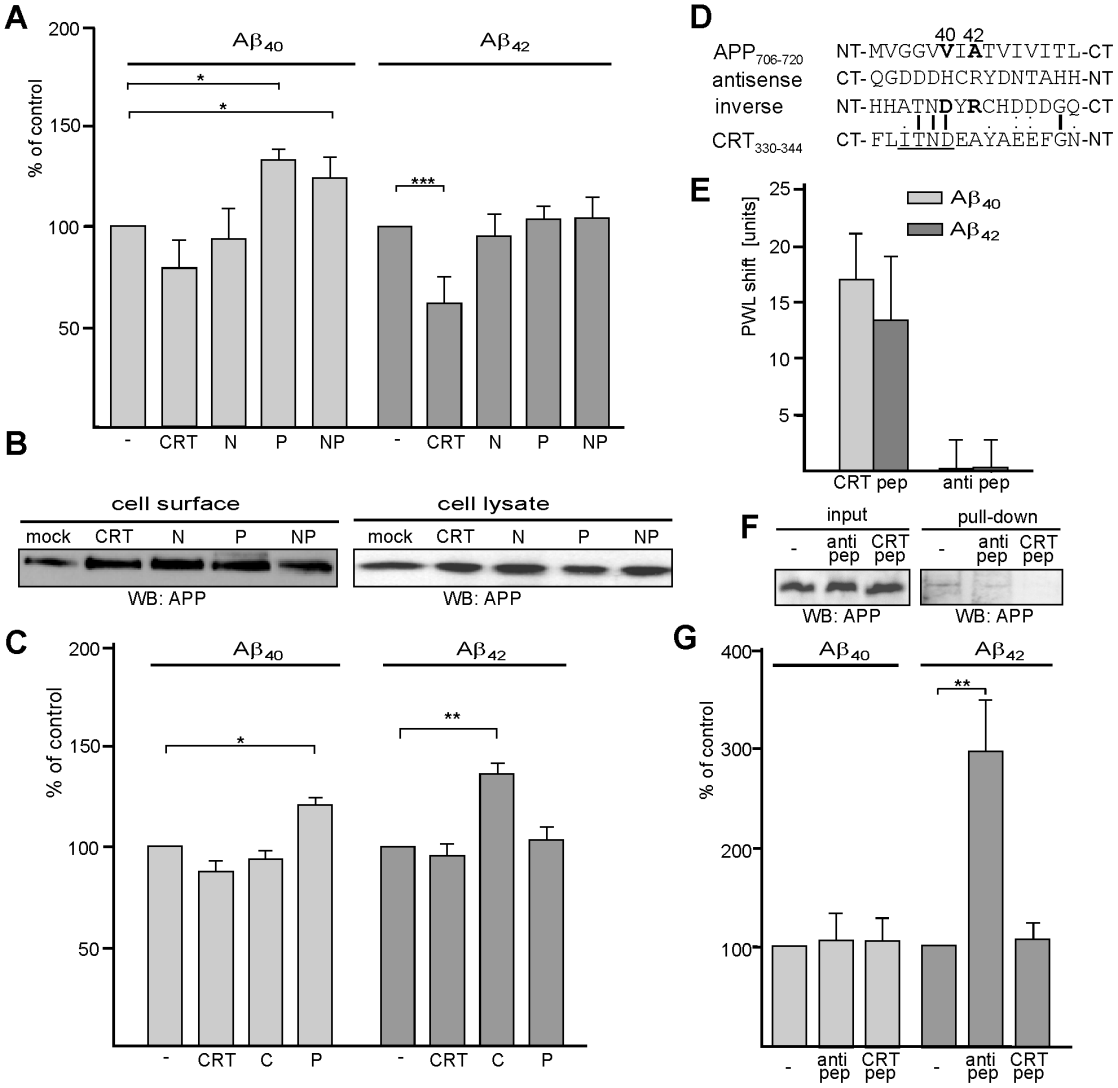
Calreticulin affects the production of Aβ_40_ and Aβ_42_. (**A, B**) CHO cells overexpressing APP were mock-transfected (mock) or transfected with constructs encoding the P-domain (P), the N-domain (N) or the N- and the P-domain (NP) of calreticulin or full-length calreticulin (CRT). (**B**) Transfected cells were subjected to cell surface biotinylation, biotinylated proteins were isolated and Western blot analysis of cell lysates and biotinylated cell surface proteins with the APP antibody A8717 was performed. (**C**) CHO cells overexpressing APP were incubated with GST-calreticulin (CRT), GST/C-domain (C) or GST/P-domain (P). (**D**) Amino acids 706–710 of APP comprising the γ-cleavage site for generation of Aβ_40_ and Aβ_42_ the sequence of the corresponding antisense peptide and the inverted sequence of the antisense peptide are shown. Similarities between the inverse antisense peptide sequence and amino acid 330–344 of calreticulin are indicated (bar, colon and dot represent identical, highly conserved and weakly conserved amino acids). NT: N-terminus; CT: C-terminus. (**E**) Label-free binding assay using substrate-coated Aβ_40_ or Aβ_42_ peptides and soluble calreticulin peptide comprising amino acids 330–344 (CRT pep) or antisense peptide (anti pep). Mean values ± SD from triplicates of a representative experiment are shown. (**F**) GST/C-domain fusion was incubated with a detergent extract of mouse brain homogenate in the absence (-) or presence of calreticulin peptide (CRT pep) or antisense peptide (anti pep). Proteins bound to the GST/C-domain were precipitated with glutathione-agarose (pull-down) and subjected to Western blot analysis with the APP antibody A8967. Aliquots taken from the samples before precipitation were used as input control. (**G**) CHO cells overexpressing APP were incubated in the absence or presence of the calreticulin peptide (CRT pep) or antisense peptide (anti pep). (**A, C, G**) Cell culture supernatants were collected and the amounts of Aβ_40_ and Aβ_42_ in cell culture supernatants were determined by ELISA. The levels obtained after mock-transfection were set to 100%. Mean values ±SEM from 3 independent experiments with biological duplicates per experiment are shown (Students t-test * p>0.05, ** p>0.005).

Since calreticulin interacts with sequences at the γ-cleavage site of APP, we investigated whether calreticulin influences the cleavage of APP by γ-secretase and thus affects Aβ production. To this aim, APP overexpressing CHO cells were transfected with constructs encoding full-length calreticulin or different calreticulin domains followed by determination of Aβ_40_ or Aβ_42_ levels in cell culture supernatants as measured by ELISA. Transfection with full-length calreticulin decreased the levels of Aβ_42_ in comparison to mock-transfection, but only slightly affected levels of Aβ_40_ (Fig. 7A). In contrast, upon transfection of constructs encoding the calreticulin P-domain or the N- and P-domains, levels of Aβ_40_ but not of Aβ_42_ in the cell culture supernatant were increased, while the levels of both Aβ_40_ and Aβ_42_ in culture supernatants of cells expressing the N-domain were unchanged relative to mock-transfection (Fig. 7A). Interestingly, transfection with the construct encoding the C-domain of calreticulin led to cell death.

Cell surface biotinylation was carried out to elucidate whether the expression of the P-domain or full-length calreticulin affected the translocation of APP to the cell surface and, thus, resulted in the observed alterations in Aβ production. Levels of APP at the cell surface and in cell lysates were not altered in cells expressing the P-domain of calreticulin or full-length calreticulin in comparison to the levels of APP observed at the cell surface of mock-transfected cells (Fig. 7B), indicating that expression of full-length calreticulin and of calreticulin domains affect the proteolytic processing of APP rather than the transport of APP to the plasma membrane.

In a next step, we addressed the question whether extracellular application of full-length calreticulin or calreticulin domains to APP overexpressing cells would affect the generation of Aβ_40_ and/or Aβ_42_. Levels of Aβ_40_ were increased only in the presence of the GST/P-domain, while levels of Aβ_42_ were only increased in the presence of the GST/C-domain (Fig. 7C), indicating that the P- and C-domains of calreticulin interfere with the proteolytic processing of APP.

### A sequence stretch in the C-domain of calreticulin mediates the interaction with the γ-cleavage site in APP and modulates the Aβ_42_ production

According to the theory of complementary hydropathy, the antisense peptide should contain the APP binding site and/or should mimic the structure of the sequence stretch in calreticulin that interacts with the γ-cleavage site in APP. Surprisingly, the antisense peptide did not show any similarity to a sequence in calreticulin. Interestingly, however, we noticed that the inverse sequence of the antisense peptide displays a significant similarity to a sequence stretch in the C-domain of calreticulin (Fig. 7D). It has been shown for a number of sequences that their structure is similar to the structure of their inverse sequences [51]. Moreover, it has been shown that distinct sequence stretches and their inverted counterparts not only have similar structures but also that they interact with binding partners in a similar manner and mediate the same functions [52]–[55]. We thus hypothesized that the sequence stretch in the C-domain of calreticulin, which shows similarity to the inverted antisense peptide, mediates the binding to the γ-secretase cleavage site within APP. To test this idea, we first performed a label-free binding assay using Aβ_40_, Aβ_42_, antisense peptide, and a calreticulin-derived peptide comprising amino acid 330–344 and the putative binding site for APP sequences. The calreticulin peptide, but not the antisense peptide showed binding to immobilized Aβ_40_ and A β_42_ (Fig. 7E). Since the recombinant GST/C-domain precipitated APP in a pull-down assay (Fig. 4), we analyzed whether the calreticulin and/or antisense peptide interferes with the binding of the GST/C-domain to APP. In the presence of the calreticulin peptide, no APP was precipitated from detergent-solubilized brain homogenate, while APP was precipitated in the absence of peptides or in the presence of the antisense peptide (Fig. 7F), indicating that the binding of the C-domain to APP is mediated by the sequence stretch comprising amino acids 330–344. Since the application of GST/C-domain to APP overexpressing cells increased the production of Aβ_42_ (Fig. 7C), we tested whether application of the calreticulin peptide has a similar effect. The level of Aβ_42_ in the cell culture supernatant was increased in the presence of the calreticulin peptide, while the level of Aβ_40_ was not altered (Fig. 7G). Importantly, the levels of Aβ_42_ and Aβ_40_ were not altered in the presence of the antisense peptide (Fig. 7G). This result suggests that the calreticulin peptide binds to the γ-secretase cleavage site in APP and that this binding interferes with the binding of endogenous calreticulin and thus with the processing of APP and generation of Aβ_42_.

### Calreticulin interacts with presenilin and nicastrin

**Figure 8:**
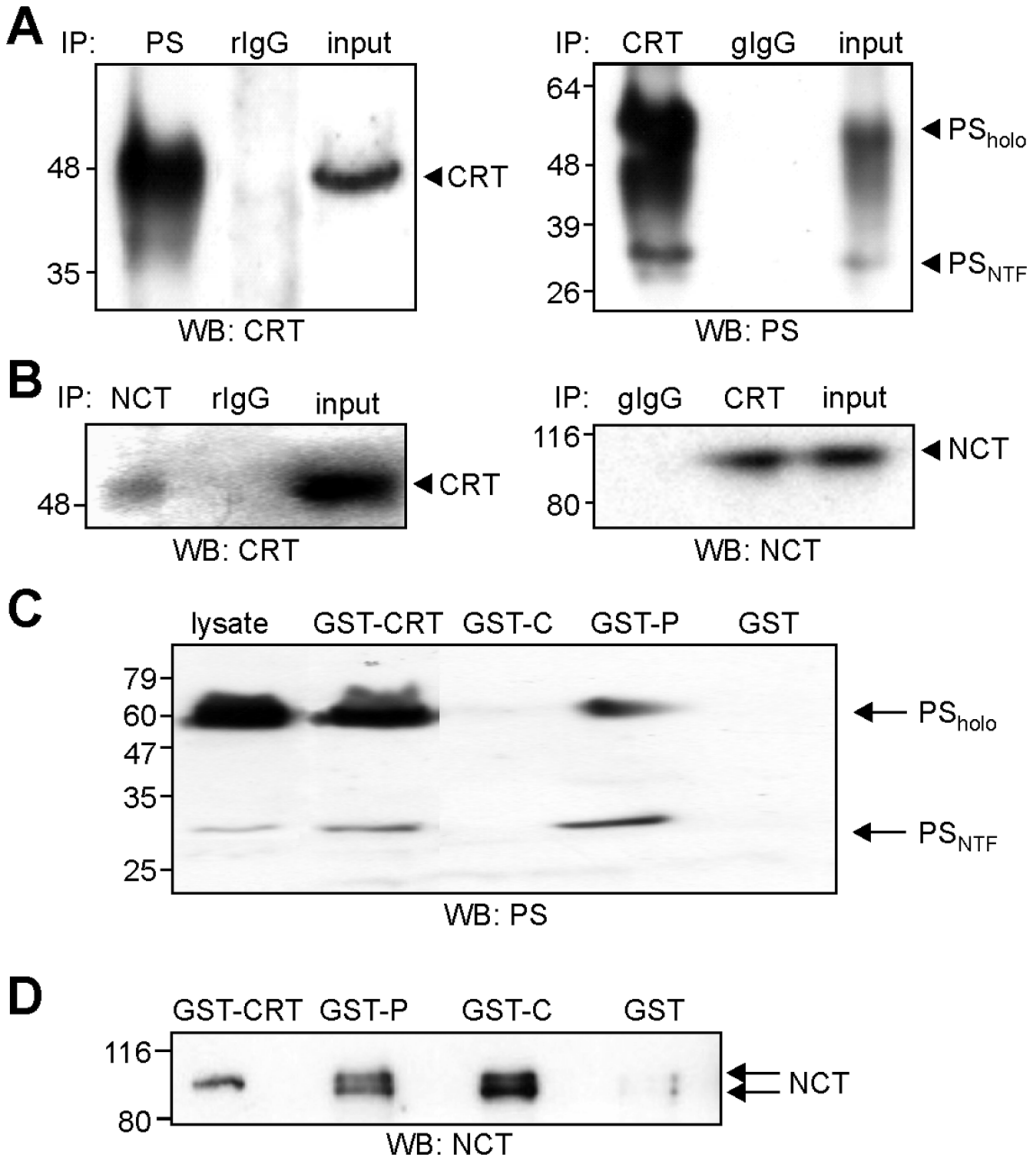
Calreticulin interacts with presenilin via its P-domain and with nicastrin via its P-and C-domain. (**A, B**) Immunoprecipitations were performed using a synaptosomal fraction and the rabbit presenilin antibody 2953 (PS; left panel) (**A**), the rabbit nicastrin antibody (NCT; left panel) (**B**), the goat calreticulin antibody C-17 (CRT; right panels) (**A, B**) and non-immune control antibodies from rabbit (rIg; left panels) and goat (gIg; right panels) (**A, B**). Immunoprecipitates and cell lysates (input) were subjected to Western blot analysis using the goat calreticulin antibody C-17 (WB: CRT; left panel) (**A, B**), the rabbit presenilin antibody `Nixon` (WB: PS; right panel) (**A**), or the rabbit nicastrin antibody (WB: NCT; right panel) (**B**). (**C, D**) Solubilized membranes of HEK cells overexpressing presenilin were incubated with GST or GST-fusion proteins comprising full-length calreticulin (GST-CRT), the P-domain (GST-P) or the C-domain (GST-C) bound to glutathione-agarose beads. Precipitated proteins were subjected to Western blot analysis using the rabbit presenilin antibody 2953 (**C**) or the rabbit nicastrin antibody (**D**). (**A-D**) The positions of uncleaved presenilin holoprotein (PS_holo_), the N-terminal presenilin fragment (PS_NTF_) and nicastrin (NCT) are indicated by arrows.

Since calreticulin and its P-domain affect the proteolytic processing of APP by γ-secretase, we investigated whether calreticulin is associated with the γ-secretase complex. Therefore, co-immunoprecipitation experiments were performed using detergent extracts of a synaptosomal fraction. Western blot analysis with a goat polyclonal calreticulin antibody showed that calreticulin is present in the presenilin immunoprecipitates (Fig. 8A). The full-length uncleaved 55 kDa presenilin and the N-terminal 28 kDa presenilin fragment were observed in the calreticulin immunoprecipitates by Western blot analysis with the presenilin antibody (Fig. 8A). When using non-immune control antibodies for immunoprecipitation, neither calreticulin nor presenilin was detectable (Fig. 8A). Since calreticulin co-immunoprecipitates with presenilin, we checked whether other subunits of the γ-secretase complex were associated with calreticulin and tested whether nicastrin, PEN-2 or APH-1 co-immunoprecipitated with calreticulin. Western blot analysis of immunoprecipitates from the synaptosomal fraction revealed weak but significant co-immunoprecipitation of calreticulin using nicastrin antibodies and, vice versa, strong co-immunoprecipitation of nicastrin when using a calreticulin antibody (Fig. 8B). Non-immune control antibodies did not co-immunoprecipitate calreticulin or nicastrin (Fig. 8B). No co-immunoprecipitation of calreticulin with APH-1 or PEN-2 was observed (data not shown). These results demonstrate an association of calreticulin with presenilin and nicastrin.

Next, we investigated whether calreticulin interacts with the γ-secretase complex at the plasma membrane. To this aim, live cultured hippocampal neurons were incubated with calreticulin antibodies against the N- or C-terminus, fixed and stained with a presenilin antibody and corresponding fluorescent-labeled secondary antibodies. Both calreticulin antibodies showed a pronounced co-staining with the presenilin antibody at the surface of cell bodies and along neurites (Fig. 6F), indicating that calreticulin interacts with the γ-secretase complex at the plasma membrane of neurons.

### The P-domain of calreticulin interacts with presenilin, whereas the P- and C-domains of calreticulin interact with nicastrin

Although the P-domain did not interact with APP (Fig. 4), expression of the P-domain in APP overexpressing cells or application of recombinant P-domain to APP overexpressing cells led to an increase in Aβ_40_ levels (Fig. 7A, C). On the other hand, co-immunoprecipitation (Fig. 8A, B) and co-localization of calreticulin (Fig. 6F) with presenilin and nicastrin indicated associations between calreticulin and presenilin and/or nicastrin. Thus, we hypothesized that calreticulin binds to presenilin and/or nicastrin and that the P-domain has a dominant-negative effect on γ-secretase mediated cleavage of APP by competing with the endogenous calreticulin for binding to presenilin and/or nicastrin. To validate this idea, we first tested whether the P-domain interacts with presenilin and/or nicastrin by a pull-down assay using a membrane fraction from presenilin overexpressing cells and GST-fusion proteins comprising full-length calreticulin or domains of calreticulin. GST/calreticulin and GST/P-domain pulled down the mature uncleaved 55 kDa presenilin and the 28 kDa N-terminal fragment of presenilin (Fig. 8C). When using the GST/C-domain and GST as controls, neither forms of presenilin were pulled down (Fig. 8C). Western blot analysis with nicastrin antibody showed one band of approximately 110 kDa when the GST fusion protein with full-length calreticulin was used for pull-down, while a double band of 100 and 110 kDa which may represent mature and immature forms of nicastrin, respectively [56], [57] was observed when the P-domain or C-domain was used for pull-down (Fig. 8D). GST alone showed no pull-down of nicastrin (Fig. 8D). These results indicate that the P-domain of calreticulin mediates the interaction with presenilin, whereas the P- and C-domain interact with nicastrin.

### The activity of γ-secretase is reduced by full-length calreticulin, but is enhanced by the P-domain of calreticulin

**Figure 9:**
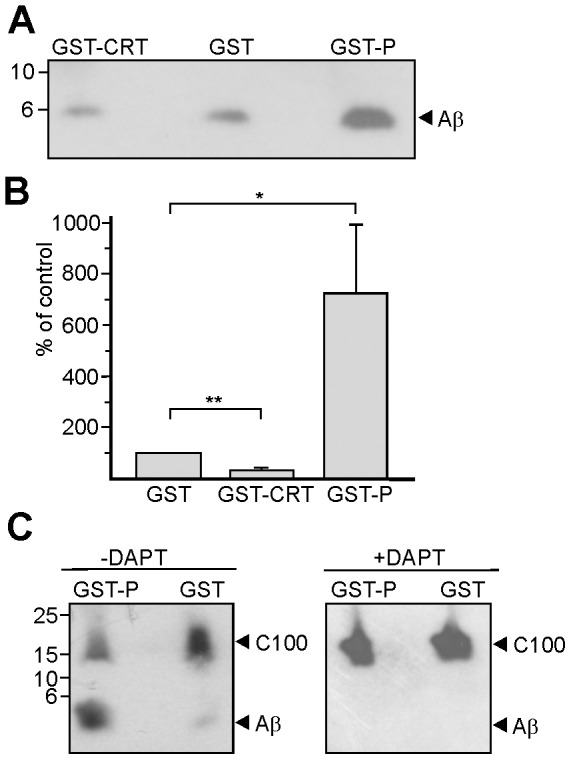
The γ-secretase activity is altered by calreticulin and the P-domain of calreticulin. Immunoprecipitated γ-secretase was incubated with C100 APP stump as substrate and GST or the GST-fusion protein with full length calreticulin (GST-CRT) or the P-domain of calreticulin (CRT-P) in the absence (**A-C**) or presence of the γ-secretase inhibitor DAPT (**C**). Aβ was detected by Western blot analysis using the rabbit antibody 2D8 (**A**) and the amount of Aβ was determined by densitometry (**B**). (**C**) Western blots showing uncleaved C100 substrate and Aβ in the presence of GST and GST-P and in the absence or presence of DAPT. Mean values ±SD are from 4 experiments (Students t-test * p>0.005, ** p>0.0005).

Since calreticulin associates with the γ-secretase complex and since expression of full-length calreticulin and the P-domain of calreticulin alter Aβ production, we tested in an *in vitro* γ-secretase activity assay whether GST/calreticulin and GST/P-domain modulate the cleavage of APP by γ-secretase. In the presence of the GST/P-domain, the amount of Aβ generated from the C100 APP stump was higher when compared to the amount observed in the presence of GST, while the amount was lower in the presence of GST/calreticulin relative to that obtained in the presence of GST (Fig. 9A). Quantification revealed an increase of Aβ levels by 722+274% in the presence of the GST/P-domain and a reduction by 68+11% in the presence of GST/calreticulin relative to the level observed in the presence of GST which was set to 100% (Fig. 9B). To verify that the enhanced cleavage of the C100 APP stump and the generation of Aβ were mediated by γ-secretase, immuno-isolation of γ-secretase and assay of γ-secretase activity assay were carried out in the absence or presence of the γ-secretase inhibitor DAPT. A small portion of the C100 APP stump (<10%) was converted to Aβ in the presence of GST, while a large portion (>70%) was cleaved in the presence of the GST/P-domain (Fig. 9C). In the presence of DAPT, no Aβ was detectable even in the presence of the GST/P-domain (Fig. 9C), indicating that calreticulin modulates γ-secretase mediated cleavage of APP via its P-domain.

## Discussion

### The binding of calreticulin to the γ-secretase cleavage site of APP is mediated by amino acids 330–344 in its C-domain

**Figure 10:**
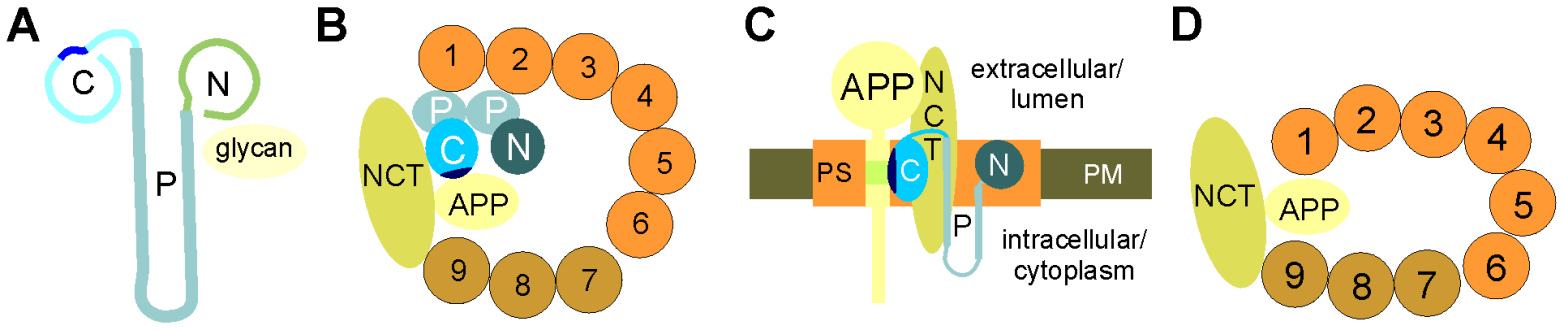
Working model for the interactions between calreticulin, APP, presenilin and nicastrin. (**A**) Schematic representation of calreticulin structure (side view): N- and C-domain are globular and the P-domain forms a hairpin-loop structure. The N- and P- domains form the recognition site for glycans. The calreticulin sequence stretch comprising amino acids 330–344 is highlighted (dark blue) (**B**) Schematic representation of interactions between calreticulin domains, APP, nicastrin (NCT) and presenilin (transmembrane domains are indicated by numbered cycles) in a bird's eye view onto the cell surface. In the water-filled cavity/pore formed by presenilin, APP interacts with the C-domain of calreticulin via amino acids 330–344 (dark blue), while nicastrin interacts with the C- and P-domain of calreticulin. The P-domain interacts with the N-terminal presenilin fragment consisting of the N-terminal transmembrane domains TMD1-TMD6 (circles in orange). The P-domain does not interact with the C-terminal presenilin fragment comprising the C-terminal transmembrane domains TMD7-TMD9 (circles in gold). Nicastrin interacts with TMD9. (**C**) Schematic representation of the proposed topology of calreticulin domains within the γ-secretase complex in the plasma membrane in a side view of the vertically cut membrane. Nicastrin (NCT) and presenilin (PS; orange box) are depicted in the plasma membrane (PM). The interaction between the amino acids 706–720 of APP within the γ-secretase cleavage sites (light green) and amino acids 334–340 of calreticulin within the C-domain (dark blue) is shown. (**D**) In the absence of calreticulin, APP can directly interact with nicastrin which functions as substrate receptor for APP as well as with domains TMD1 and TMD9 of presenilin.

In a previous study, expression of calreticulin at mRNA and protein levels were found to be reduced by 30–50% in brains from patients with AD compared to brains from neurologically normal individuals [32]. Moreover, antibodies against calreticulin stained damaged neurons in brain tissue from AD patients and the numbers of cells stained by calreticulin antibodies and the intensity of calreticulin immunostaining were lower than in normal control brains [32]. The levels of BIP (binding immunoglobulin protein; also known as glucose-regulated protein 78), which is, like calreticulin, an ER chaperone that binds to APP [58], [59] were not altered in brains of AD patients relative to control brains [32]. These findings suggested that calreticulin is associated with the pathogenesis of AD, while other ER chaperones appear to be not or less involved. Since calreticulin has been shown in a complex with BIP and Aβ in the cerebrospinal fluids of normal individuals [31], it is conceivable that calreticulin is also involved in preventing aggregation of Aβ in AD brains. These observations also suggested that calreticulin interacts with APP and Aβ *in vivo*. Here, we identified calreticulin as an APP interaction partner that binds to sequences at the γ-secretase cleavage site and showed that calreticulin binds to APP within a sequence stretch containing the γ-secretase cleavage site and to Aβ_40_ and Aβ_42_. The binding of calreticulin to APP does not dependent on Ca^2+^ or N-glycans on APP. This finding excludes that binding of the Ca^2+^-dependent C-type lectin calreticulin [47], [48], [60] to APP is mediated by N-linked carbohydrate structures on APP. In a previous study using APP overexpressing cell lines [30], the interaction between APP and calreticulin was neither disrupted in the presence of the Ca^2+^-specific chelator EGTA nor in the presence of inhibitors of oligosaccharide trimming. These findings also agree with our observation that APP and calreticulin do not interact through a lectin-like mechanism. Since the N- and P-domains are responsible for the carbohydrate recognition [28] (Fig. 10A) and since we showed that the C-domain of calreticulin mediates the binding to APP, binding of calreticulin to APP does not occur in a lectin-like manner.

The sequence stretch comprising amino acids 330–344 in the C-domain of calreticulin mediates the binding to APP. This sequence stretch displays similarity to the inverted antisense peptide with conservation of a Thr-Asn-Asp (TND) motif.

### Calreticulin forms a complex with APP at the cell surface

Co-immunostaining of APP and calreticulin at the cell surface of cultured hippocampal neurons indicates that APP and calreticulin are indeed associated at the cell surface. The concomitant increase of cell surface levels of APP and calreticulin observed by cell surface biotinylation in cells overexpressing mutated presenilin indicates that APP reaches the plasma membrane together with calreticulin and that both proteins are present at the cell surface as a complex. Based on the findings by us and others indicating that calreticulin is present in extracellular compartments [21]–[26], [29], we find it conceivable that extracellular calreticulin could also interact with the γ-secretase cleavage site of APP at the plasma membrane. On the other hand, co-immunoprecipitation of calreticulin with immature APP from a synaptosomal fraction indicates an interaction of APP with calreticulin already in the ER during maturation and trafficking of APP. As seen in our study, an association of calreticulin with immature as well as the mature APP has also been shown by co-immunoprecipitation of calreticulin from APP overexpressing cell lines [30]. In this previous study, it was shown that APP and calreticulin interact and that this interaction was detectable at pH 7.5, decreased at pH 6.5 and was not detectable at pH 5.5. These results suggest that the interaction between APP and calreticulin takes place under pH conditions prevailing in the ER and early *cis*-Golgi compartment or at the plasma membrane, but not under the acidic pH condition prevailing in the *trans*-Golgi or endosomal compartments. Since the generation of the APP intracellular domain from a GFP-tagged C99 occurs exclusively at the plasma membrane [14], it is very likely that cleavage of APP by γ-secretase occurs at the cell surface and not in the ER, Golgi or endosomal compartments. This notion is supported by our finding that extracellular application of the C- or P-domain of calreticulin or the calreticulin peptide interferes with the generation of Aβ_40_ and Aβ_42_. In addition, calreticulin seems to be tightly associated with or embedded in the plasma membrane, since it remains associated with synaptosomal membranes after treatment with alkaline pH and EDTA and is released only after detergent treatment. Moreover, immunostaining of calreticulin at the cell surface of live hippocampal neurons with antibodies against its N- or C-terminus indicate that calreticulin is accessible from the outside of the cell and is, thus, present in or at the plasma membrane. Furthermore, extracellular application of the recombinant GST/C- and GST/P-domains to APP overexpressing cells affects APP processing, indicating that they compete with the domains of endogenous calreticulin at the plasma membrane and impair the function of endogenous calreticulin in a dominant-negative manner. This dominant-negative impact on APP proteolysis is even more pronounced when the calreticulin peptide was applied to APP overexpressing cells, implying that the small 14mer is most effective in blocking the function of endogenous calreticulin at the plasma membrane. In contrast, extracellularly applied recombinant GST/calreticulin does not affect the APP processing indicating that it is not functional in this configuration, possibly due to improper folding, sterical hindrance by GST or inaccessibility of domains.

Calreticulin also binds to Aβ in a time- and concentration-dependent manner, being enhanced in the presence of Ca^2+^ and optimal at pH 5 [61]. These observations suggest that calreticulin binds also to Aβ and APP in an acidic environment. However, although this Ca^2+^-dependent binding appears to be different from the Ca^2+^-independent interaction between APP and calreticulin observed in the present study, the observation that calreticulin binds to Aβ agrees with our findings that calreticulin binds to Aβ and to sequences flanking the γ-secretase cleavage site of APP and overlapping with those of Aβ.

### Calreticulin associates with presenilin and nicastrin and modulates γ-secretase activity

A novel finding of our study is that calreticulin interacts with presenilin and nicastrin, members of the γ-secretase complex (Fig. 10B, C) as shown by co-immunoprecipitation. Other subunits of the γ-secretase complex, PEN-2 and APH-1, did not co-immunoprecipitate with calreticulin, suggesting that calreticulin is not an integral constituent of the γ-secretase complex and that it associates with the γ-secretase complex only transiently. This notion is supported by the observation that calreticulin is not detected in a highly purified preparation of the γ-secretase complex [38]. The interaction between calreticulin and presenilin depends exclusively on the P-domain, while the interaction of calreticulin with nicastrin is mediated by the C- and P-domains (Fig. 10B, C). Since presenilin does not carry N-glycans, a carbohydrate-mediated interaction between calreticulin and presenilin can be excluded. Furthermore, it is also unlikely that the interaction of calreticulin with nicastrin is due to the binding of calreticulin to glycans on nicastrin, because the interaction with carbohydrates requires both the N- and P-domains of calreticulin [36], [60] (Fig. 10A), while nicastrin is associated only with the P-domain.

The interaction of calreticulin with presenilin and/or nicastrin affects the cleavage of APP by γ -secretase. Expression of calreticulin in APP overexpressing cells reduces the generation of the neurotoxic Aβ_42_, while expression of the P-domain, which binds to presenilin and/or nicastrin and competes with the binding of endogenous calreticulin, increases the production of Aβ_40_. Similarly, in the presence of recombinant calreticulin less Aβ is generated from the C100 APP stump by the γ-secretase complex, while more Aβ is generated in the presence of the P-domain. Moreover, application of the recombinant P-domain of calreticulin to live APP overexpressing cells leads to an increase in Aβ_40_ production, while application of the calreticulin C-domain increases Aβ_42_ levels. Application of recombinant calreticulin does not affect Aβ_40_ or Aβ_42_ levels. These findings imply that recombinant calreticulin is not functional to reduce Aβ production, whereas the recombinant C- and P-domains compete with the domains of endogenous calreticulin leading to alterations in Aβ production. The dominant-negative effect of the C-domain and the enhanced production of neurotoxic Aβ_42_ cause cell death as we observed upon overexpression of the C-domain in APP expressing cells. Based on these findings, we propose that calreticulin protects APP from cleavage by the γ-secretase complex, most likely via binding of the P-domain to presenilin and/or nicastrin (Fig. 10B) as well as binding of the C-domain to APP and/or nicastrin. This feature of calreticulin may have important implications in AD pathology. Since our results indicate that calreticulin co-localizes with APP and presenilin at plasma membrane of hippocampal neurons and modulates the APP cleavage by presenilin, it is conceivable that calreticulin is present within the catalytic pore formed by the γ-secretase complex with its N- and C-terminal domains exposed to the extracellular space (Fig. 10B, C). The putative location in the catalytic pore would allow calreticulin to interact with APP, presenilin and nicastrin (Fig. 10B) and to regulate the processing of APP by the presenilin. Ablation of calreticulin, reduction of calreticulin levels or interference with calreticulin's binding to APP enables APP to interact directly with presenilin and the other components of the γ-secretase complex (Fig. 10D) leading to increased proteolytic cleavage of APP, to enhanced Aβ production and to aggregation of Aβ, contributing to the amyloidogenic aspect of AD pathology. Interestingly, reduced calreticulin mRNA and protein levels and enhanced levels of neurotoxic Aβ have been found in brains of patients with AD [32]. This *in vivo* finding and our *in vitro* observations that calreticulin binds to APP, Aβ_40_ and Aβ_42_, presenilin and nicastrin and that it reduces the production of Aβ_40_ and Aβ_42_ provide strong evidence that calreticulin regulates the γ-secretase-mediated processing of APP *in vivo*, raising the possibility that calreticulin may be a target in preventing an important aspect of AD pathology.
